# Study on the Psychological States of Olfactory Stimuli Using Electroencephalography and Heart Rate Variability

**DOI:** 10.3390/s23084026

**Published:** 2023-04-16

**Authors:** Tipporn Laohakangvalvit, Peeraya Sripian, Yuri Nakagawa, Chen Feng, Toshiaki Tazawa, Saaya Sakai, Midori Sugaya

**Affiliations:** 1 College of Engineering, Shibaura Institute of Technology, Tokyo 135-8548, Japan; 2 Research & Development Division, S.T. Corporation, Tokyo 161-0033, Japan

**Keywords:** olfactory stimuli, electroencephalography (EEG), heart rate variability (HRV)

## Abstract

In the modern information society, people are constantly exposed to stress due to complex work environments and various interpersonal relationships. Aromatherapy is attracting attention as one of the methods for relieving stress using aroma. A method to quantitatively evaluate such an effect is necessary to clarify the effect of aroma on the human psychological state. In this study, we propose a method of using two biological indexes, electroencephalogram (EEG) and heart rate variability (HRV), to evaluate human psychological states during the inhalation of aroma. The purpose is to investigate the relationship between biological indexes and the psychological effect of aromas. First, we conducted an aroma presentation experiment using seven different olfactory stimuli while collecting data from EEG and pulse sensors. Next, we extracted the EEG and HRV indexes from the experimental data and analyzed them with respect to the olfactory stimuli. Our study found that olfactory stimuli have a strong effect on psychological states during aroma stimuli and that the human response to olfactory stimuli is immediate but gradually adapts to a more neutral state. The EEG and HRV indexes showed significant differences between aromas and unpleasant odors especially for male participants in their 20–30s, while the delta wave and RMSSD indexes showed potential for generalizing the method to evaluate psychological states influenced by olfactory stimuli across genders and generations. The results suggest the possibility of using EEG and HRV indexes to evaluate psychological states toward olfactory stimuli such as aroma. In addition, we visualized the psychological states affected by the olfactory stimuli on an emotion map, suggesting an appropriate range of EEG frequency bands for evaluating psychological states applied to the olfactory stimuli. The novelty of this research lies in our proposed method to provide a more detailed picture of the psychological responses to olfactory stimuli using the integration of biological indexes and emotion map, which contributes to the areas such as marketing and product design by providing insights into the emotional responses of consumers to different olfactory products.

## 1. Introduction

In the modern information society, people are constantly exposed to stress due to several problems such as work environment, overwork, long overtime hours, sleep deprivation, and depression. According to an occupational safety and health survey conducted by the Ministry of Health, Labor and Welfare of Japan in 2018, 58.0% of all workers (59.9% males and 55.4% females) reported having high stress caused by their current job and working life [1]. Moreover, the stress experienced by workers has continued to increase over the past five years, suggesting that it will continue to expand.

Aromatherapy has been attracting attention as one of the ways to relieve stress using aroma. Aromatherapy uses aromatic substances or essential oils extracted from plants, such as herbs, to promote mental and physical health. The Aroma Environment Association of Japan conducted a survey on the consuming behavior of aroma, asking, “Do you ever use aroma in your daily life?” As a result, 41% answered “frequently/occasionally”, indicating that aromas are used in daily life [2].

Aromatherapy has many applications, from physical and mental relaxation to therapeutic applications to relieve some symptoms. Many studies have been conducted on aromatherapy. Thirty percent of these studies have focused on nursing care to relax and relieve tension, followed by improvement of sleep disturbance and sleep–wake rhythm, pain relief, and reduction of fatigue and anxiety [3].

To clarify the usefulness of aromatherapy, it is necessary to evaluate the effect of aromas quantitatively and instantly. Therefore, this research proposes a method to measure biological signals affected by aromas and other olfactory stimuli to observe changes in human psychological states. A previous study reported that a simple electroencephalogram (EEG) sensor with the excellent temporal resolution is promising for obtaining psychological states in response to arousal level [4]. Moreover, the previous study illustrated that it is possible to evaluate the state of the autonomic nervous system in terms of pleasantness and unpleasantness from heart rate measurement [5]. Therefore, Ikeda et al. proposed a method to evaluate emotions and psychological states in accordance with a well-known Russell’s circumplex model of affect by using the combination of EEG and heart rate variability (HRV) indexes to evaluate arousal and valence levels, respectively [6]. The method has been applied to evaluate psychological states toward human–robot interaction and visual stimuli. In addition, they proposed an “emotion map” as a simple yet useful method to visualize the positioning and transition of human emotions and psychological states in corresponding to a widely used Russell’s circumplex model of affect [7]. However, it has not yet been employed to evaluate the psychological states toward olfactory stimuli like aroma.

Therefore, this study aims to investigate the relationship between biological indexes and the psychological effect of aromas using two types of biological signals, EEG and HRV. In this study, we performed not only the statistical analysis of EEG and HRV indexes but also integrated those two indexes to the emotion map, which can provide more comprehensive understanding of the effects of olfactory stimuli on the emotional states. This research contributes to several areas such as marketing and product design, by providing insights into the emotional responses of consumers to different olfactory products. It also has implications for the development of new treatments for help in regulating emotions and improving mental health outcomes.

## 2. Related Studies

### 2.1. Methods for Estimating Psychological States

Various methods have been employed in studies related to evaluating human psychological states and emotions. Subjective evaluation methods such as questionnaires and self-reports are commonly used because of many advantages such as simplicity and low cost. However, human psychological states are implicit and usually expressed unpredictably, which are difficult to evaluate just by using subjective evaluation methods [8]. To compensate for those difficulties, many studies employed objective evaluation methods such as measuring biological signals [9], which can provide quantitative output and catch unconscious and immediate human responses [10]. 

There are various biological signals such as EEG, photoplethysmogram (PPG), electrocardiogram (ECG), eye movements, etc. The EEG is utilized in a wide range of fields, including psychology, neuroscience, medicine, and engineering because it provides a non-invasive and cost-effective way to measure the electrical activity of the brain in comparison to other brainwave sensors to measure brain activity [11,12]. One of the main advantages of EEG is its high temporal resolution, meaning it can detect changes in brain activity with high precision and accuracy in real-time [13]. This makes it a valuable tool in studying cognitive processes such as attention, perception, and memory. EEG can also be used to investigate the effects of various stimuli on the brain, including sensory input like smell, sound, and light, or cognitive tasks like problem-solving or decision-making [14,15]. In addition, it has been used to recognize emotions and represent cognitive functions such as distraction, fatigue, and concentration [16,17,18,19]. Alarcao and Fonseca reported an emotion estimation survey using EEG signals to detect unconscious emotions from EEG signals [20]. Fraiwan et al. constructed a machine learning model to estimate the enjoyment and visual interest of participants experiencing museum content using 8-channel EEG signals [21].

In addition, several studies used HRV obtained from ECG signal, PPG signal, etc. [22,23]. The HRV is utilized in various studies because it provides an accurate and non-invasive measure of autonomic nervous system (ANS) function, which is responsible for regulating many involuntary physiological processes in the body, such as heart rate, blood pressure, digestion, and respiration [24]. HRV measures the variation in time between successive heartbeats, which reflects the balance between the two branches of the ANS—the sympathetic and parasympathetic branches [25]. HRV has been studied in a variety of fields, including psychology, neuroscience, sports science, and medicine. In psychology and neuroscience, HRV is used to measure stress, anxiety, and emotion regulation [26,27]. Moreover, it has been used to recognize and classify various psychological states such as joy, anger, sadness, happiness, relaxation, and excitement [28,29,30,31]. Thus, HRV is a valuable tool for researchers and clinicians to assess autonomic function and its relationship with various physiological and psychological outcomes.

Not only one specific biological signal but also multimodal biological signals have been used to estimate people’s emotions, psychological states, or other cognitive processes [32,33,34]. The related studies usually collect and analyze multiple types of biological signals such as EEG, ECG, electrodermal activity (EDA), and respiratory signals [35]. By combining different types of biological signals, researchers can gain a more comprehensive understanding about human response to various stimuli, allowing for a more accurate and robust analysis of emotional and cognitive states. Ikeda et al. combined EEG and HRV indexes to evaluate unconscious emotions by proposing to map those indexes to a well-known Russell’s circumplex model of affect, a psychological model which represents emotions on a two-dimensional axis, i.e., arousal and valence [6]. Suzuki et al. employed the same method to construct a classification model for emotion estimation and achieved high classification accuracy by integrating feature selection algorithms [36].

### 2.2. Relationship between Olfactory Stimuli and Psychological States

Various literature has provided scientific evidence for the benefits of aroma on physical health. Human olfaction is anatomically related to the autonomic nervous system. The autonomic nervous system regulates the activities of the circulatory, digestive, and respiratory systems under the direction of the brain, and the sense of smell is closely related to the control of the body through brain commands. Olfactory stimuli are transmitted to the central nervous system through olfactory receptors in olfactory cells in the olfactory epithelium. However, recent studies have shown that the olfactory epithelium contains not only olfactory receptors but also adrenergic receptors in sympathetic nerve endings and muscarinic receptors in parasympathetic nerve endings. Therefore, it is assumed that the autonomic nervous system is activated and inhibited by olfactory stimulation in healthy subjects [37].

Furthermore, in the medical field, there is a medical treatment called “medical aromatherapy”, which is distinguished from ordinary aromatherapy. Medical aromatherapy is a complementary and alternative medicine that uses essential oils to treat diseases and relieve symptoms [38]. However, it is also used more broadly in nursing and caregiving and for therapeutic purposes. In the nursing field, it can relieve various symptoms in addition to its central role of reducing the anxiety of patients and their families. In the nursing care field, essential oils are actively used not only for their sedative effects but also for their ability to stimulate the central nervous system. For example, Kanzaki et al. conducted an experiment with healthy participants sniffing the fragrances. They reported that the activity of the autonomic nervous system increased by the effect of rose-like odor (pleasing odor) and greatly enhanced by feces-like odor (bad odor) [39]. Furthermore, Tanida et al. reported that the odor of grapefruit activated the sympathetic nervous system and described various effects of aroma on the autonomic nervous system [40].

The literature has shown that olfactory stimuli, such as aromas, promote the activation of the human through the brain and autonomic nervous system, and that investigating the effect of aromas would help reveal how to apply them to daily life appropriately.

### 2.3. Relationship between Olfactory Stimuli and Biological Signals

Different olfactory stimuli can cause different reactions to biological signals. Some types of aromas, such as lavender and ylang-ylang, are reported to affect the decrease of the brain’s activity. Masago et al. examined the effects of essential oils on autonomic nervous system function using ECG, skin blood flow, galvanic skin response (GSR), and blood pressure, focusing on lavender, rosemary, and citronella, the three most frequently used essential oils, and reported that a refreshing aroma like rosemary temporarily stimulated the sympathetic nervous system [41]. Moreover, aromas with individual preferences, such as citronella, tend to have more complicated effects on the autonomic nervous system. They further examined the effects of lavender, chamomile, sandalwood, and eugenol aromas using 12-channel (F3, F4, C3, C4, P3, P4, O1, O2, F7, F8, T5, and T6) EEG sensor [41]. From each channel, θ (4–8 Hz), α1 (8–10 Hz), α2 (10–13 Hz), and β (13–30 Hz) were analyzed. A significant decrease in α1 was observed in the left parietal and left posterior regions during the 10 s immediately following aroma inhalation. This study showed significant changes in α1 activity during aroma inhalation. The α-wave activity usually corresponded to some sensory stimuli in addition to olfactory stimuli [42].

Duan et al. examined the effects of lavender on the autonomic nervous system and brain activity using positron emission tomography (PET) and HRV indexes [43]. There was no significant change in the overall brain activity. Still, there were some changes of activity in some brain regions, specifically, the left posterior cingulate gyrus and the right temporal lobe. In terms of HRV, significant changes in LF and HF were observed. This suggests that lavender may have the effect of inhibiting sympathetic nerves first and then increasing vagal nerves.

In recent years, several studies on the relationship between olfactory stimuli and biological signals have been conducted using commercially available sensors. Babini et al. investigated the correlation between heart and brain responses collected from 5-channel EEG and ECG sensors in response to four types of odors. They suggested that the odor complexity and variation cause an increase in the complexity of EEG and HRV [44]. Jiang et al. conducted a study on the psychological effects of fragrant flowers by measuring EEG, pulse rate, and blood pressure [45]. The results showed that exposure to fragrant flowers induced relatively better psychological effects compared to non-fragrant ones, which implies the use of fragrant indoor plants to enhance the quality of the environment and promote human health. Lekamge et al. investigated the psychophysiological effects of eight different aromas under a short-term stressor through ECG and skin conductance level, and reported that cedarwood, strawberry, green tea, apple, and citrus ginger had inhibitory effects on cardiac sympathetic nervous system elevation and parasympathetic nervous system suppression [46]. Zhao et al. conducted a study to explore the relationship between different types of tree peony fragrances and human psychophysiological responses combining EEG and HRV [47]. The results showed that inhaling the fragrances led to lower heart rates and increased parasympathetic nervous activities as well as an increase in the EEG’s low alpha, high alpha, and theta waves.

Several studies have already investigated the relationship between olfactory stimuli and biological signals, but some limitations remain. They mainly focused on the change of biological signals or the overall change of psychological states such as an increase of relaxation caused by aromas, however, the measurement of degrees of such psychological states toward the olfactory stimuli have not been focused. Therefore, our research extending from those limitations is intended to widen the understanding and practical method to evaluate human psychological states toward olfactory stimuli using biological signals.

## 3. Experimental Method

### 3.1. Olfactory Stimuli

Six air fresheners regarded as aromas (i.e., citrus, lavender, soap, woody, fruity, and floral) from commercial products of S.T. Co., Ltd. (Tokyo, Japan), and one reagent with an unpleasant odor were used as olfactory stimuli. All seven stimuli were presented by smelling the odor solution in a paper strip. The details of the stimuli are shown in Table 1.

### 3.2. Measuring Instruments

**Figure 1:**
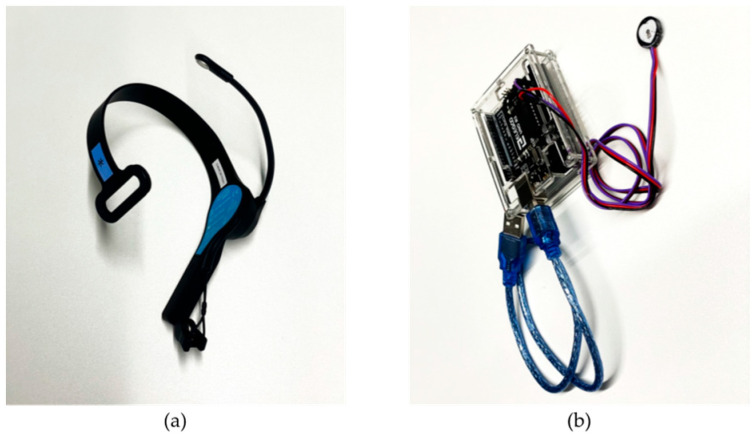
Biological sensors used in the experiment: (**a**) EEG sensor; (**b**) pulse sensor connected to Arduino board.

We employed two types of biological sensors to evaluate the psychological states while the olfactory stimuli were exposed to the participants. The details are as follows:

- EEG sensor (Figure 1a): We placed an electrode near the left frontal lobe, namely, the AF3 channel as defined by the International 10–20 EEG system, using Mindwave Mobile 2 manufactured by NeuroSky. It is a simple, low-invasive single-channel EEG sensor with a sampling rate of 512 Hz. The output from this EEG sensor is acquired approximately once per second.
- Pulse sensor (Figure 1b): We attached the pulse sensor to an index finger using a reflection-type PPG pulse sensor manufactured by SparkFun Electronics that works in combination with an Arduino kit for data transmission to the computer. It is a low-cost, fast-response, and simple sensor with a sampling rate of 500 Hz. The output from this pulse sensor is acquired approximately once per second, along with the EEG output.

### 3.3. Experimental Setup and Procedure

**Figure 2:**
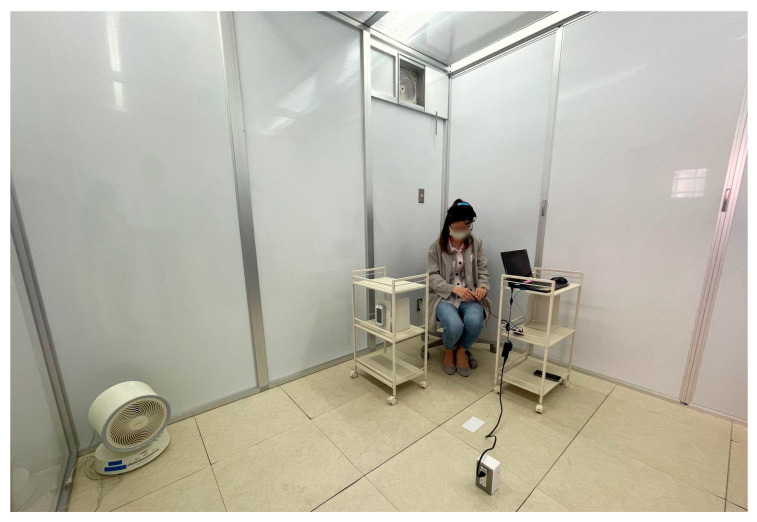
Experiment room.

The experiment was performed in a quiet and well-ventilated room at room temperature. The room has been confirmed to have no unpleasant scents that may disturb the experiment. The experiment room is shown in Figure 2.

The participants were instructed not to wear any perfume on the day of the experiment. Before starting the experiment, they were asked to wash their hands up to the elbows with unscented soap.

**Figure 3:**
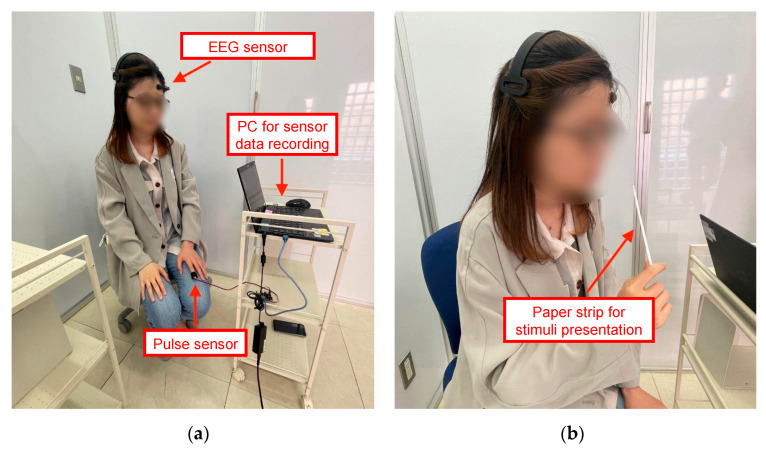
Experimental scene (**a**) when attaching sensors and (**b**) during stimulus presentation.

The experimental scene is shown in Figure 3. The experiment was conducted by the following procedure:

1. Explain the experiment and obtain written informed consent for experiment cooperation;
2. Attach pulse sensor and EEG sensor and start sensor recording;
3. Perform pre-resting condition by sitting still for 1 min;
4. Perform stimulus presentation condition for 1 min;
5. Answer the questionnaire about the stimulus;
6. Perform post-resting condition by sitting still for 1 min;
7. Repeat steps 3 to 6 until all stimuli are presented;
8. Stop sensor recording and finish the experiment.

The six aroma stimuli were presented in random order among the participants, while the unpleasant odor was always presented as the last stimulus for all participants with a time limitation of 30 s. Note that this paper presents only results from biological signals, while the questionnaire results will be used in our future study. The above procedure has been validated through our pilot study [48] to test the experimental protocol designed by following the olfactory measurement method issued by the Minister of the Environment, Japan [49]. Based on the pilot study, we confirmed that the psychological effect on EEG and HRV can be observed as well as the issues and areas for improvement in the experimental procedure identified.

### 3.4. Participants

We recruited a total of 62 participants, who are males and females in their 20s to 50s. The details of the number of participants for each gender and generation are shown in Table 2. The research was performed with the approval of and in accordance with the Shibaura Institute of Technology Institutional Review Board. Written informed consent was obtained from all participants.

## 4. Data Analysis Method

### 4.1. EEG and HRV Indexes

We obtained several EEG parameters from the EEG sensor developed by NeuroSky Inc. [50]. In this research, Attention and Meditation are used as the indexes of arousal and drowsiness, respectively, based on previous research by Ikeda et al. [6]. Since they are regarded as an opposite pole (arousal vs. drowsiness), we calculated their difference by subtracting the two parameters (denoted as Attention−Meditation for the rest of this paper) and used it as an EEG index to indicate the arousal level. The high value of Attention−Meditation indicates a high arousal level and vice versa. In addition, we obtained the values of the power spectrum, which calculates the strength of each frequency band of EEG indexes (Table 3).

Previous studies showed the usefulness of Attention and Meditation as indexes for the evaluation of human states in various kinds of applications [6,53,54]. However, these indexes are known to be the values calculated directly from the original algorithm developed by NeuroSky [50], which made it difficult to interpret the results using these indexes. Thus, it is necessary to focus on investigating more transparent and standardized methods for evaluating psychological states from EEG data.

Due to such limitations, we also employed other EEG indexes from different frequency bands that can also be obtained from the NeuroSky’s EEG sensor, which are delta (δ) wave, theta (θ) wave, and the ratio of high beta (Highβ) and high alpha (Highα) wave. We used several frequency bands to support the interpretation of psychological states that cannot be conducted by Attention−Meditation alone. 

Since EEG signals can be very noisy due to many factors, we first applied the moving average as a smoothing method with a window size of 15 s, similar to the method performed by Suzuki et al. [36]. Finally, we obtained the moving average of the index such as the ratio of Highβ and Highα (denoted as MA Highβ/Highα for the rest of this paper) as an EEG index to indicate the arousal level. The high value of MA Highβ/Highα (in other words, Highβ is the denominator) indicates a high arousal level as the participant feels alert, excited, and agitated. On the other hand, the low value of MA Highβ/Highα (in other words, Highα is the denominator) indicates a low arousal level as the participant feels relaxed but still aroused and concentrated. It has been reported that the Attention index emphasizes β band and the Meditation index emphasizes α band, according to the previous study [55]. Therefore, the ratio of Highβ and Highα is considered an important arousal index to compare to Attention−Meditation in our data analysis.

Moreover, θ and δ waves were also employed as we assumed that they might have a certain relationship with olfactory stimuli due to brain activation in low-frequency bands such as relaxation during aroma inhaling. The higher values of these indexes indicate a higher relaxing state [56]. In this study, we excluded gamma (γ) wave from our analysis because it usually has high muscle artifacts due to its small amplitude and is not widely used compared to other slower brainwaves [57].

For the HRV index, we employed pNN50 and the root mean square of successive differences between normal heartbeats (RMSSD). Among several HRV indexes, these two indexes were selected because they have been successfully used in experiments with the ultra-short-term period of one minute or less. The pNN50 was proposed as an index to evaluate valence or pleasant levels in the previous study [6]. The RMSSD is typically used as a stress-relax index [58]. On the other hand, though various other HRV indexes may have the potential to evaluate the psychological states, they require over 5 min or 24 h of measurement [35] which does not suit our experiment in which the olfactory stimuli are presented to participants in a short time. Therefore, these two HRV indexes were considered appropriate for the analysis in our study.

In total, we used the combination of EEG and HRV indexes for the evaluation of psychological states toward the olfactory stimuli. The EEG indexes (i.e., Attention−Meditation, MA Highβ/Highα, θ wave, and δ wave) were used for evaluating the arousal level. The HRV indexes (i.e., pNN50 and RMSSD) were used for evaluating the valence level.

### 4.2. Data Normalization Method

Since the data collected from each participant can be vastly varied due to the individual differences in biological signals, we normalized the data before performing further analysis by subtracting the average of each index by the average baseline obtained from the resting period of each participant. Therefore, our further analysis considered only the change of the biological indexes during each olfactory stimulus from the baseline condition. The reason that we selected the resting period as baseline is that this condition was conducted at the beginning of the experiment, and thus, it has no effect from any stimuli. Therefore, it was used to indicate the neutral state of the participants.

The change $(E_{X}$) was obtained using the Equations (1) and (2). $B_{avg}$ denotes the average of the biological index during the baseline or resting period (from the 0th to the nth second), $E_{X}$ denotes the difference of the biological index X during the exposure of each olfactory stimulus E, where i denotes the nth second of stimulus exposure time, and X denotes the biological signal parameters (i.e., Attention, Meditation, Highβ, Highα, or pNN50). We subtracted the average value of baseline $\left(B_{avg}\right)$ from the *i*th stimulus $\left(E_{x}{}_{i}\right)$. This yields the normalized form of biological index $\left(E_{X}\right)$, which is the change in the biological index from the baseline measurement in response to each stimulus.

(1) $$B_{avg}=\frac{1}{N}\overset{n}{\underset{i=1}{\sum }}B_{i}$$

(2) $$E_{X}=E_{x_{i}}-B_{avg}$$

### 4.3. Data Grouping 

For the analysis, we grouped the data based on the following criteria:

- Stimulus exposure time
  ○ The entire exposure time (60 s for all aromas, 30 s for IVA)
  ○ Half of the exposure time (first half, latter half)

- Participant gender and generation
  ○ Male, 20s–30s (age range 20–39 years old) (N = 15)
  ○ Male, 40s–50s (age range 40–59 years old) (N = 15)
  ○ Female, 20s–30s (age range 20–39 years old) (N = 17)
  ○ Female, 40s–50s (age range 40–59 years old) (N = 15)

### 4.4. Data Visualization by Emotion Map

Ikeda et al. proposed an “emotion map” method for visualizing the transition of biological indexes acquired from EEG and HRV onto the two-dimensional coordinates of arousal and valence based on the basic concept of emotion estimation by Russell’s circumplex model of affect. The emotion map displays the EEG index values of the central nervous system and the HRV values of the autonomic nervous system on the same two-dimensional coordinate system, enabling an intuitive understanding of the states of olfactory stimulation. 

In this study, we employed the emotion map to observe the psychological response reflected on biological indexes. The Attention−Meditation and the MA Highβ/Highα were used to represent the arousal level (Y-axis). The pNN50 was used to represent the valence level (X-axis). The ranges of biological indexes on the emotion map were calculated as follows:

- Attention−Meditation: According to NeuroSky’s definition, Attention and Meditation have values ranging from 0 to 100 [50]. Since we calculated them by subtraction, the range becomes −100 to 100 with the origin point of 0.
- MA Hβ/Hα: Since the MA Hβ/Hα is a ratio value, the minimum value is 0. The maximum value is scaled by the maximum value of all data to include all data on the emotion map. In this paper, we set the maximum value as 2, which covers all data with balanced scaling between negative and positive ranges on the axis.
- δ wave: Since we used its raw data, its values represent the magnitude of EEG wave and have no units. Thus, it is meaningful when being considered as relative quantity and temporal fluctuations. We used this index as supporting information to interpret the Attention−Meditation. Therefore, we did not limit the range of this index like other indexes.
- pNN50: The range is 0 to 1, with the origin calculated from the average resting condition at the start of the experiment. Thus, the origin is different for each emotion map, making the scale between the left and right sides of the origin point unbalanced. Note that we did not use RMSSD to plot the emotion map because it is typically used for the stress-relax index [58], which is slightly different from the pNN50 proposed as the valence index. Thus, we only used it in the statistical analysis to support the interpretation of pNN50.

**Figure 4:**
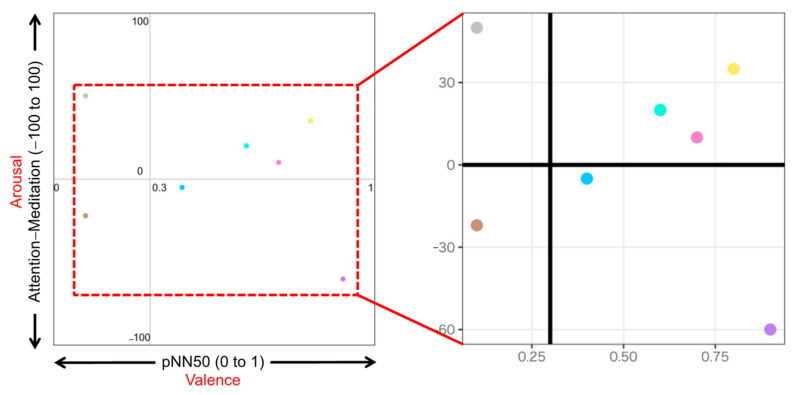
Example of an emotion map using Attention−Meditation as Arousal index and pNN50 as Valence index. The dots with different colors indicate the different olfactory stimuli.

Figure 4 illustrates an example of an emotion map using Attention−Meditation as the arousal index and pNN50 as the valence index. The left plot illustrates the full-scale emotion maps. In this example, the arousal scales from −100 to 100 with 0 as a fixed origin point, and the valence scales from 0 to 1 with 0.3 as the origin point calculated from the average of resting condition of the participant data used in that emotion map. The right plot illustrates the zoomed version of the left emotion maps for a closer observation of the plots with a reduced scale. Similar emotion maps can be created in the same way by changing the arousal index to other EEG indexes (i.e., MA Hβ/Hα or δ wave) with different ranges. There is no change for pNN50 as the valence index for the creation of any emotion map.

## 5. Experimental Results and Discussion

### 5.1. Statistical Comparison among Olfactory Stimuli

We conducted the statistical analysis of the data according to the grouping described in Section 4.3. Four groups of participants were divided by gender and generation. The biological indexes were calculated by dividing them into the first and latter half of the stimulus exposure time. Only the changes (normalized data) were used in the analysis. The statistical analysis method employed was a repeated measure ANOVA, with a post hoc pairwise comparison with no adjustment of *p*-values. The repeated measures ANOVA is used when the same group of participants is measured on two or more occasions, which allows us to analyze the changes within the same group over time while reducing the effects of individual differences and increasing the power of the analysis since the same participants are being compared [59]. In this case, the same group of participants was measured on seven occasions (i.e., seven olfactory stimuli) as a factor.

We plotted the average changes of biological indexes among the seven olfactory stimuli divided into the first half and latter half of the stimulus exposure time for each of the four participant groups. From the post hoc pairwise comparison, we obtained significant differences between some pairs of stimuli, as indicated by the red horizontal line on the figures. The detailed analysis results of the EEG and HRV indexes are described next.

#### 5.1.1. Analysis of EEG Indexes

**Figure 5:**
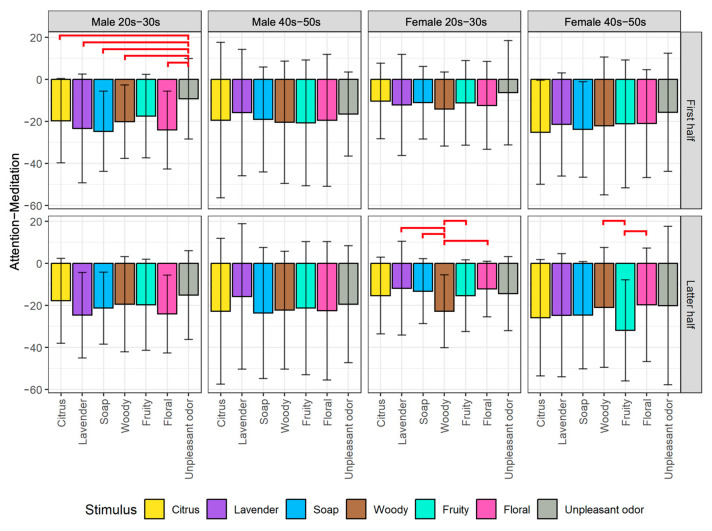
Comparison of changes in Attention−Meditation among stimuli by gender and generation groups divided into the first half (top) and latter half (bottom) of stimulus exposure time. The red lines indicate significant differences between pairs of stimuli.

For the results of Attention−Meditation as shown in Figure 5, from the first half of the stimulus exposure time, there are significant differences in Attention−Meditation values for the male 20s–30s group when comparing unpleasant odor to almost all aromas, i.e., citrus (*p* < 0.05), lavender (*p* < 0.01), soap (*p* < 0.01), woody (*p* < 0.05), and floral (*p* < 0.01). This result indicates that the unpleasant odor had a higher arousal effect than aromas on males in the younger generation group.

From the latter half of the stimulus exposure time, there are significant differences in Attention−Meditation values for the female 20s–30s group when comparing woody to lavender (*p* < 0.01), soap (*p* < 0.05), fruity (*p* < 0.05), and floral (*p* < 0.01). These findings suggest that woody is an aroma-type olfactory stimulus that had a stronger effect on meditation in the younger generation of female participants. Additionally, there are significant differences in Attention−Meditation values for the female 40s–50s group when comparing fruity to woody (*p* < 0.05) and floral (*p* < 0.05), indicating that fruity is an aroma-type olfactory stimulus that had a stronger effect on meditation for the older generation of female participants.

These findings suggest that the effect of olfactory stimuli tends to differ by gender and generation. Therefore, to properly evoke certain psychological states through aromas, it might be necessary to consider the gender and age of the consumers.

**Figure 6:**
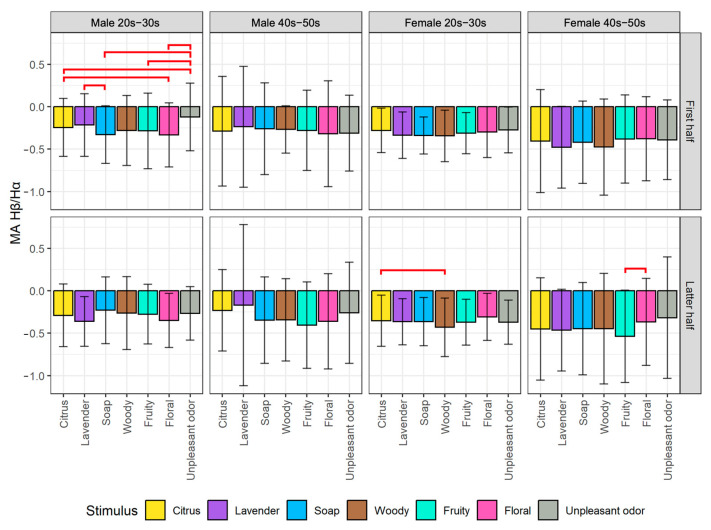
Comparison of changes in MA Hβ/Hα among stimuli by gender and generation groups divided into the first half (top) and latter half (bottom) of stimulus exposure time. The red lines indicate significant differences between pairs of stimuli.

For the results of MA Hβ/Hα as shown in Figure 6, all participant groups tend to be more relaxed when being exposed to all olfactory stimuli, as indicated by the changes of MA Hβ/Hα toward the negative values. From the first half of the stimulus exposure time, there are significant differences in MA Hβ/Hα values for the male 20s–30s group when comparing unpleasant odor to citrus (*p* < 0.05), soap (*p* < 0.05), fruity (*p* < 0.05), and floral (*p* < 0.001), as well as when comparing citrus vs. floral (*p* < 0.05) and lavender vs. soap (*p* < 0.05). This result indicates that the unpleasant odor had a higher arousal effect than aromas on males in the younger generation group, which resembles the result of Attention−Meditation. In addition, the result reveals additional findings between the aromas suggesting a higher effect on relaxation for soap over lavender and floral over citrus, which provide a hint for the suitable aromas to evoke relaxation for younger males.

From the latter half of the stimulus exposure time, there is a significant difference in MA Hβ/Hα values for the female 20s–30s group when comparing citrus vs. woody (*p* < 0.05). The result indicates that citrus leads to a slightly higher arousal level while woody leads to a higher alpha level (i.e., higher concentration). On the other hand, there is a significant difference in MA Hβ/Hα values for the female 40s–50s group when comparing fruity and floral (*p* < 0.05), which resembles the result of Attention−Meditation. The result indicates that floral leads to a higher arousal level while fruity leads to a higher alpha level (i.e., higher concentration).

**Figure 7:**
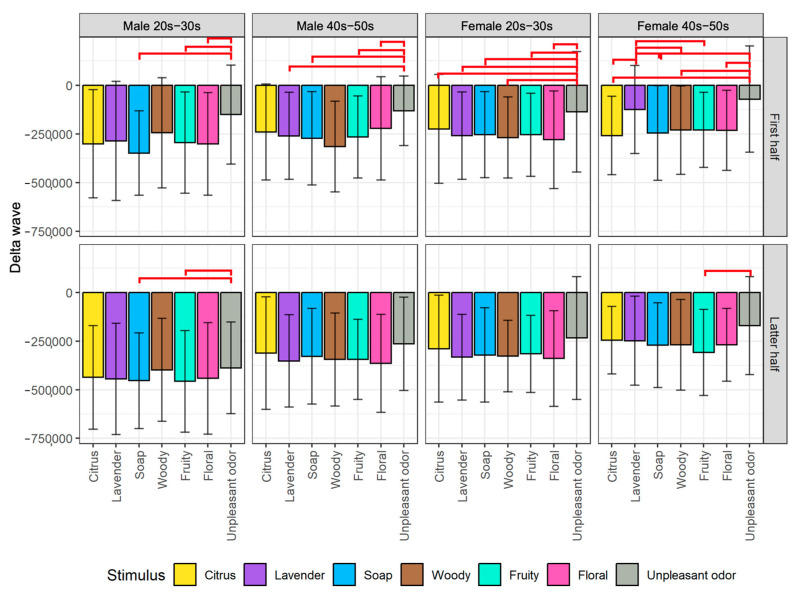
Comparison of changes in δ wave among stimuli by gender and generation groups divided into the first half (top) and latter half (bottom) of stimulus exposure time. The red lines indicate significant differences between pairs of stimuli.

For the results of δ wave as shown in Figure 7, there is a significant decrease in δ wave when participants were exposed to an aroma stimulus in the first half of exposure time compared to baseline measurements. The δ wave is associated with deep sleep, and a decrease in δ wave could potentially indicate a state of increased alertness or arousal of the brain activity. We also found that the changes in δ wave differed significantly depending on the type of olfactory stimuli. For the male 20s–30s group, unpleasant odor produced significantly different δ wave responses compared to soap (*p* < 0.05), fruity (*p* < 0.05), and floral (*p* < 0.05) for the first half of stimulus exposure time. For the male participants in their 40s and 50s, the unpleasant odor produced significantly different δ wave responses compared to lavender (*p* < 0.05), soap (*p* < 0.05), fruity (*p* < 0.05), and floral (*p* < 0.05).

For female participants, we observed different patterns of δ wave responses depending on the generation and the type of olfactory stimuli. For the female participants in their 20s and 30s, unpleasant odor produced significantly different δ wave responses compared to all other stimuli. For the female 40s–50s group, unpleasant odor produced significantly different δ wave responses compared to citrus (*p* < 0.05), woody (*p* < 0.05), and floral (*p* < 0.05). Additionally, we found significant differences in δ responses between lavender and other aromas, including citrus (*p* < 0.05), soap (*p* < 0.05), woody (*p* < 0.05), and fruity (*p* < 0.05) aromas for female participants in their 40s and 50s.

A few significant differences were seen in the second half of the aroma exposure time. This means that the olfactory stimuli tend to have greater impact on brain activity during the early stages of exposure, suggesting that the duration of the olfactory stimulation play a key role in the strength and duration of its effects. Moreover, individual differences in response to aroma stimuli could also be a factor in the differences observed.

Our study suggests that aroma stimuli can significantly impact δ wave activity in the brain, and that the specific effects can vary depending on factors such as age and type of olfactory stimuli. The significant decrease in δ wave activity observed in our study is consistent with previous research demonstrating the effects of odor on brain activity and could potentially indicate increased alertness or arousal in response to the olfactory stimuli. Furthermore, our findings highlight the importance of considering individual differences in response to olfactory stimuli, such as age and gender, in order to more accurately assess the effects of olfactory stimuli on brain activity.

#### 5.1.2. Analysis of HRV Indexes

**Figure 8:**
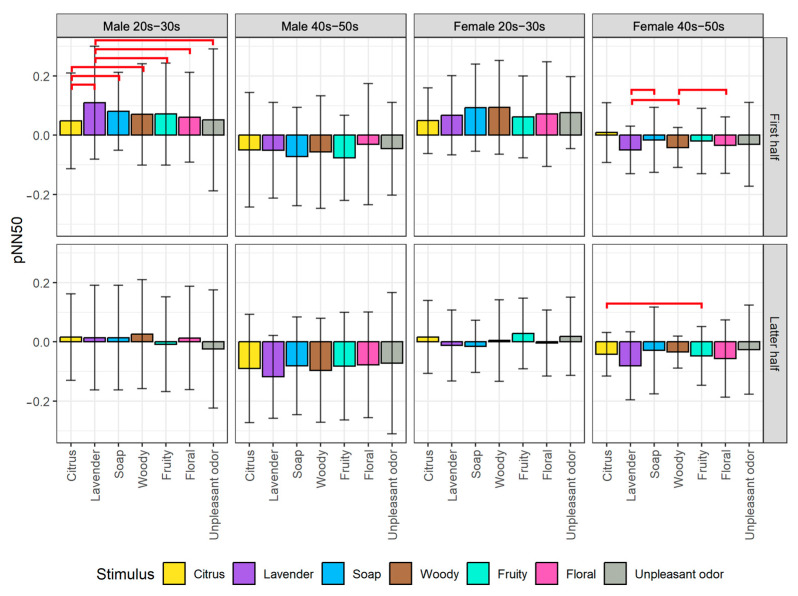
Comparison of changes in pNN50 among stimuli by gender and generation groups divided into the first half (top) and latter half (bottom) of stimulus exposure time. The red lines indicate significant differences between pairs of stimuli.

For the results of pNN50 as shown in Figure 8, from the first half of the stimulus exposure time, there are significant differences in pNN50 values for the male 20s–30s group when comparing lavender to citrus (*p* < 0.05), fruity (*p* < 0.05), floral (*p* < 0.01), and unpleasant odor (*p* < 0.05). The result indicates that lavender is an aroma-type olfactory stimulus with a stronger influence on the parasympathetic nervous system, as observed by the most significant change in the pNN50 value. On the other hand, there are significant differences in pNN50 values for this participant group when comparing citrus to soap (*p* < 0.05) and woody (*p* < 0.05). On the other hand, this result indicates that citrus is an aroma-type olfactory stimulus with a stronger influence on the sympathetic nervous system, as observed by the lowest change of the pNN50 value. A stimulus such as citrus, which can be associated with energizing and refreshing sensations, may activate the sympathetic nervous system, leading to a decrease in HRV, which is a sign of sympathetic activity. So, it is inferred that lavender could be used to evoke a state of relaxation while citrus could be used to evoke an energizing and refreshing state for younger males.

In contrast, for the female 40s–50s group, there were significant differences in pNN50 values when comparing lavender to soap (*p* < 0.05) and woody (*p* < 0.05) as well as when comparing woody to floral (*p* < 0.05). This result indicates that lavender may have a stronger influence on parasympathetic activity than soap and woody for older females. Additionally, there is a significant difference in pNN50 values for this participant group between citrus and fruity (*p* < 0.05) in the latter half of the stimulus exposure time. The result suggests that fruity may not have as strong of an influence on the parasympathetic nervous system as citrus aroma does after inhaling for a while for older females.

**Figure 9:**
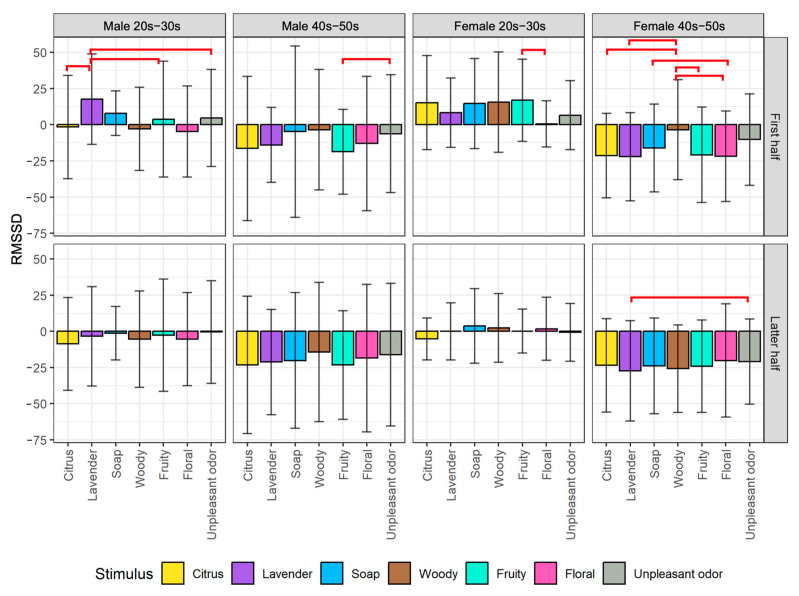
Comparison of changes in RMSSD among stimuli by gender and generation groups divided into the first half (top) and latter half (bottom) of stimulus exposure time. The red lines indicate significant differences between pairs of stimuli.

For the results of RMSSD as shown in Figure 9, there are significant differences between several pairs of olfactory stimuli in the first half of stimulus exposure time. On the other hand, there is almost no difference in the latter half, which is similar to the result observed by the changes in pNN50 (Figure 8). The results indicate that during the first half of the exposure time, lavender had a significant effect (increased from baseline measurement) on the male 20s–30s group. This is demonstrated by a statistically significant difference between lavender and citrus (*p* < 0.05), fruity (*p* < 0.05), and unpleasant odor (*p* < 0.05). The increase in RMSSD observed in the study may indicate an increase in vagal tone and parasympathetic nervous system activity, as RMSSD is a measure of vagal tone. This suggests that exposure to lavender scent may promote relaxation and stress reduction in young male individuals.

Another interesting finding is that woody as an olfactory stimulus influenced a slight decrease in RMSSD in the female 40s–50s group, as compared to their baseline measurement. This decrease was found to be significantly different from the changes observed when exposed to scents of citrus (*p* < 0.05), lavender (*p* < 0.05), fruity (*p* < 0.05), and floral (*p* < 0.05), all of which also resulted in a decrease in RMSSD when compared to baseline. These results suggest that the woody may have a different effect on the parasympathetic nervous system activity in comparison to other olfactory stimuli used in the study. The decrease in RMSSD when compared to baseline measurement could indicate the stress response to these stimuli. 

The results of the study showed that different scent stimuli had varying effects on the parasympathetic and sympathetic nervous systems of participants, depending on their gender and age group. For younger male participants, lavender had a stronger influence on the parasympathetic nervous system, while citrus had a stronger influence on the sympathetic nervous system. In contrast, for older female participants, lavender had a stronger influence on parasympathetic activity than soap and woody, while citrus had a stronger influence on the parasympathetic nervous system than fruity scents. Furthermore, exposure to woody resulted in a slight decrease in RMSSD in older female participants, indicating a potential stress response. These findings suggest that different scent stimuli may be used to evoke specific states of relaxation or stimulation in individuals, depending on their age and gender.

### 5.2. Emotion Maps

**Figure 10:**
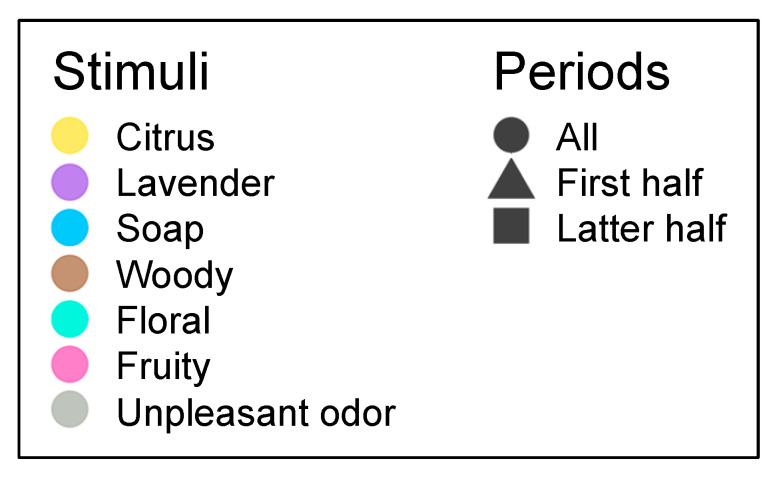
Legend for emotion map.

Based on the visualization method described in Section 4.4, we plotted the emotion maps using a combination of biological indexes. The legend of all emotion maps is shown in Figure 10. Seven types of stimuli are indicated by colors. Three types of stimulus exposure time are indicated by shapes: circle (all), triangle (first half), and square (latter half). The emotion map for each participant group is shown in two versions: the full-scale version to observe the overall psychological states on the left side of the figures and the zoom version to closely observe the difference among the stimuli on the right side of the figures.

**Figure 11:**
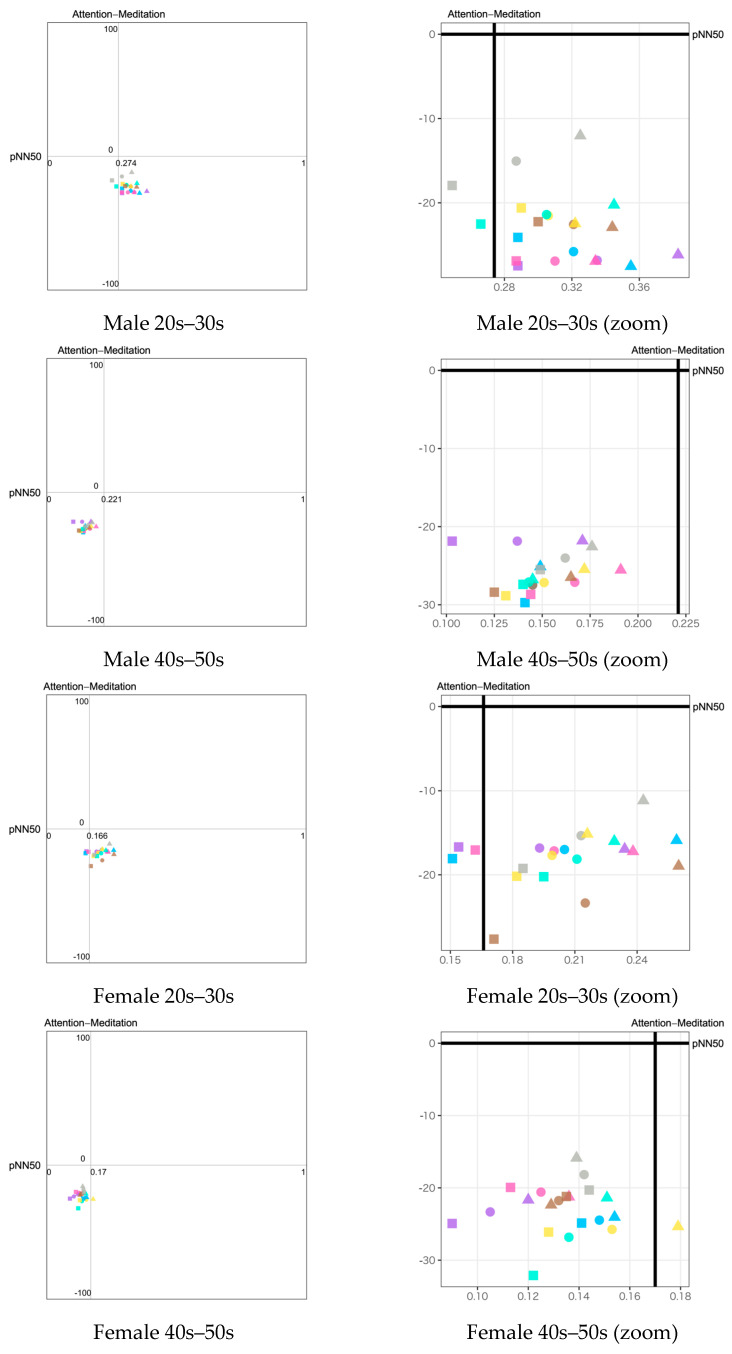
Emotion maps using pNN50 and Attention−Meditation divided by gender and generation. The left figures show the full-scale emotion maps. The right figures show the zoomed version of the emotion maps on the left for a closer observation of the plots. The dots with different colors indicate the different olfactory stimuli as shown in the legend (Figure 10).

**Figure 12:**
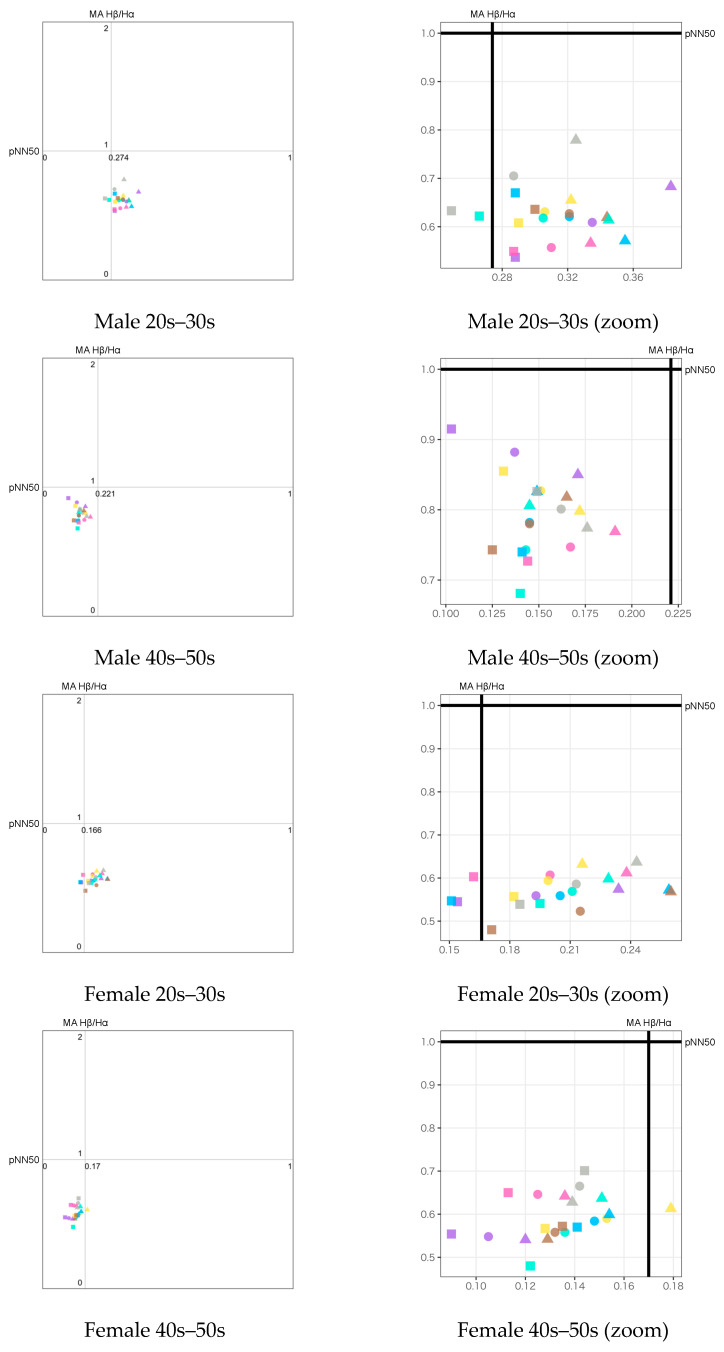
Emotion maps using pNN50 and MA Hβ/Hα divided by gender and generation. The left figures show the full-scale emotion maps. The right figures show the zoomed version of the emotion maps on the left for a closer observation of the plots. The dots with different colors indicate the different olfactory stimuli as shown in the legend (Figure 10).

**Figure 13:**
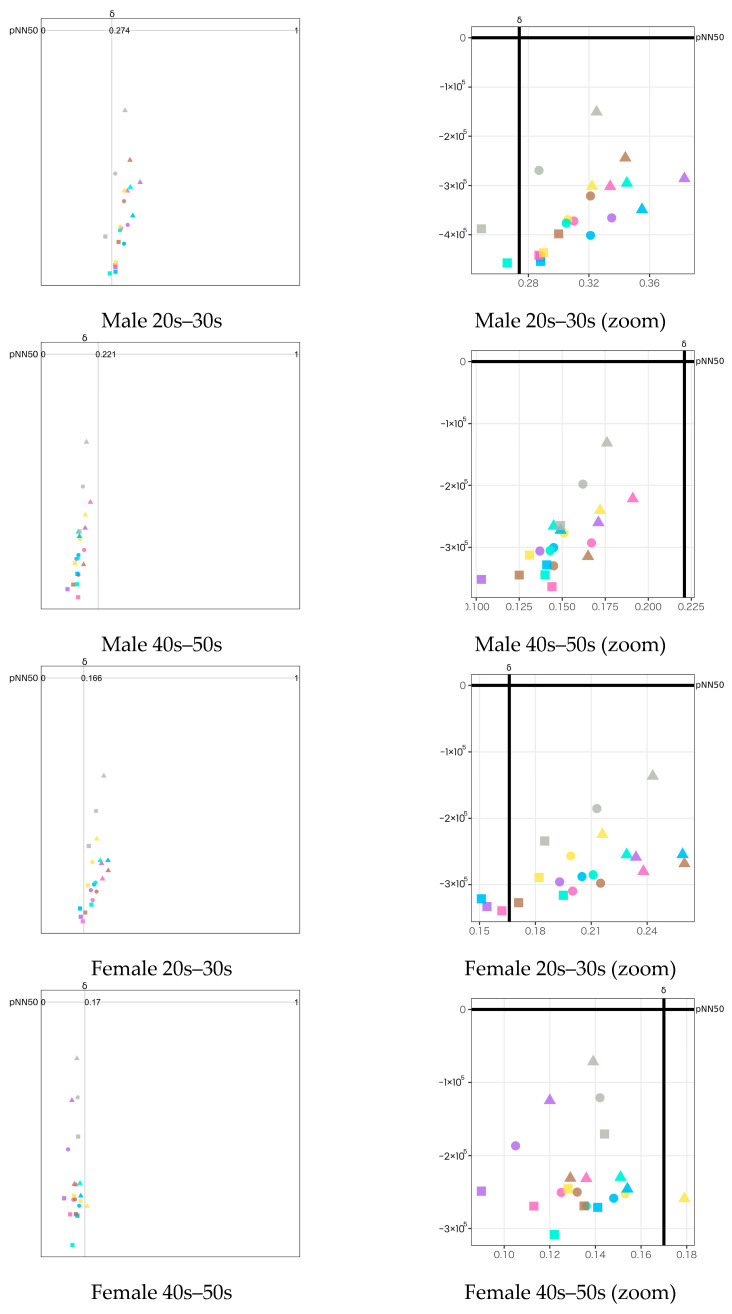
Emotion maps using pNN50 and δ wave divided by gender and generation. The left figures show the full-scale emotion maps. The right figures show the zoomed version of the emotion maps on the left for a closer observation of the plots. The dots with different colors indicate the different olfactory stimuli as shown in the legend (Figure 10).

Three types of emotion map with different combinations of HRV and EEG indexes to estimate valence and arousal levels are listed as follows:

- pNN50 (Y-axis) vs. Attention−Meditation (X-axis) (Figure 11);
- pNN50 (Y-axis) vs. MA Hβ/Hα (X-axis) (Figure 12);
- pNN50 (Y-axis) vs. δ wave (X-axis) (Figure 13).

For each of the above combinations, four emotion maps were created for the four groupings by gender and generation. For each emotion map, we plotted the average of each index calculated during three different stimulus exposure times: all, the first half, and the latter half. 

The following results are observed from the emotion maps by pNN50 vs. Attention−Meditation (Figure 11), pNN50 vs. MA Hβ/Hα (Figure 12), and pNN50 vs. δ wave (Figure 13).

- The emotion maps for both young male and female groups show low arousal and high valence levels (LAHV) near the lower right quadrant.
- The emotion maps of both older male and female groups show low arousal and low valence levels (LALV) near the lower left quadrant.

## 6. Discussion

Based on the results obtained by EEG indexes for evaluating arousal level (i.e., Attention−Meditation (Figure 5) and MA Hβ/Hα (Figure 6)), we found that the results for the first half of stimulus exposure time for the male 20s–30s group are similar. This suggests that young males are particularly sensitive to rapid changes in aroma stimuli compared to other participant groups. Furthermore, the results obtained by δ (Figure 7), especially for the first half for all four participant groups show that the aromas and the unpleasant odor can be divided more clearly. According to the results of δ, it shows the strong potential as an arousal index to classify between aromas and unpleasant odor. As additional information for detailed observation, we also provide time series plots of δ waves for each of the participant groups by gender and generation in Appendix A.

From the results of EEG indexes (i.e., Attention−Meditation, MA Hβ/Hα, and δ) that we used to indicate arousal level, all of them show similar tendency that the values were below 0 when any of the olfactory stimuli including the unpleasant odor were presented (Figure 5, Figure 6 and Figure 7). These are the values after subtracting by the value of the resting condition, meaning that the arousal levels during the stimulus conditions are lower than that of the resting condition. There are several reasons that might be able to explain such results. Firstly, aromas are generally known to increase a relaxing state especially those that people are familiar with, and thus, it is reasonable that the arousal level during the aroma condition is less than that of the resting condition. However, the same result was also obtained from the unpleasant odor, which was considered contradictory. We consider that this might be caused by two following factors: (1) Though we used the unpleasant odor, the liquid concentration might not be strong enough to increase the arousal level. In addition, the stimulus was presented for only 30 s while other aroma stimuli were presented for 60 s, and thus, the time period was too short to have a large effect on the psychological state, and (2) During the resting condition, we did not ask the participants to close their eyes. Additionally, the resting condition was conducted at the beginning of the experiment, which might cause the participants to feel excited and nervous. These uncontrollable factors might increase the arousal level as shown in the results.

Based on the results obtained by pNN50 as the HRV index for evaluating valence level (Figure 8), some pairs of the stimuli show differences in pNN50 for the first half of the stimulus exposure time, especially for young males. On the other hand, there is almost no difference in pNN50 for the latter half, suggesting that olfactory stimuli’s effect on the autonomic nervous system was notably stronger at the beginning and lasted as short as 30 s for the aromas or 15s for the unpleasant odor. Similar results were obtained from RMSSD for the latter half, which indicates that the psychological states became more stable (Figure 9). This could be because the human body adapts to the olfactory stimuli over time, causing a decrease in the perceived intensity of the stimuli and a decrease in the biological effect of the stimuli. On the other hand, RMSSD tends to be more distinguished for some olfactory stimuli for the first half. We need to further investigate how to employ RMSSD for further analysis on an emotion map, which remains as future work.

Based on the results shown on the emotion maps (Figure 10, Figure 11, Figure 12 and Figure 13), we obtained the findings as described below:

- For valence level (pNN50 on X-axis), the emotion maps of the younger generation show a tendency toward parasympathetic nerve dominance or relaxation. On the other hand, the emotion maps of the older generation show a tendency toward sympathetic nerve dominance. These results suggest that the younger generation tends to be more relaxed, while the older generation tends to be more nervous when they smell aromas. For both younger and older generations, genders tend to have less influence on psychological states.
- For arousal level (Attention−Meditation or MA Hβ/Hα on Y-axis), all of the emotion maps show that the values are low. This indicates that the values of Meditation and Hα tend to be larger regardless of any aromas or grouping. According to the literature survey, higher Hα is associated with a more relaxed mental state, which governs overall mental coordination, calmness, alertness, and mind–body integration. These results suggest that all participants may be relaxed and well-focused while smelling the aroma. For the δ wave, the values show certain differences among the olfactory stimuli. Thus, this index might be effective in classifying aromas and unpleasant odor.
- Results between Attention−Meditation and MA Hβ/Hα are consistent. However, further analysis is difficult because Attention and Meditation can only be obtained from NeuroSky’s original algorithm. Instead, MA Hβ/Hα is considered a helpful index because it allows detailed analysis based on different frequency bands of Hβ and Hα, and yet yielded the same result as Attention−Meditation. However, some results of δ wave show a different trend, which indicates we need to further investigate how to appropriately employ it on the emotion map.

Initially, we proposed to use MA Hβ/Hα as the arousal index in the emotion map. In that case, only two of the several EEG frequency bands were used, Hβ and Hα. Our target was to observe the psychological states of a person aroused by olfactory stimuli and then focus on a more detailed analysis of arousal level such as Hβ as highly focused and Hα as low focused. In addition, the lower EEG frequency bands, such as θ and δ waves, which have lower frequencies than α waves, are considered to be worth investigating whether they can be used as indexes to evaluate the drowsiness or microsleep state. From the analysis of these indexes, we found that δ wave has the potential to be used to interpret the results on the more relaxed states evoked by olfactory stimuli.

**Figure 14:**
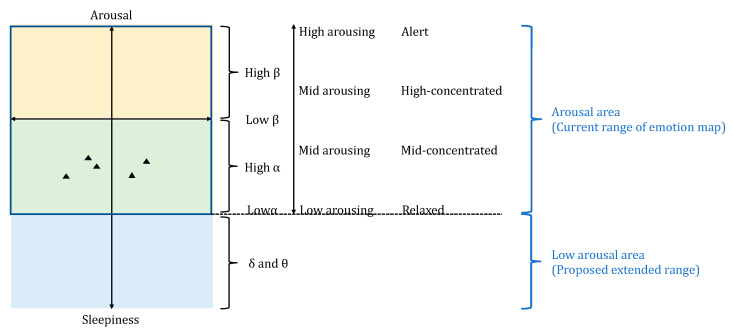
Proposed new range of arousal level in an emotion map expanding to low arousal area to visualize psychological states affected by olfactory stimuli. The triangles indicate the olfactory stimuli plotted on emotion map.

Based on the above findings of the emotion map, we propose a new range of arousal level (Y-axis), taking into account the low arousal area by using low EEG frequency bands, such as θ and δ waves, to ensure a relaxed state of the brain is included in the interpretation of psychological states as shown in Figure 14. A detailed analysis of this proposed emotion map will be performed in our future study.

From the comparison between our study and previous studies that evaluated the psychological effect of olfactory stimuli using commercially available biological sensors, we obtained similar results from EEG and HRV analysis. However, previous studies [44,45,46,47] focused only on the psychological effect on the emotions expected by olfactory stimuli, such as the relaxing state after inhaling aromas. However, our study extended the interpretation of psychological effects through EEG and HRV indexes and applied them to the emotion map in corresponding to the existing emotion model like Russell’s circumplex model of affect. With our method, the psychological states can be explained in more detail through the quantification of arousal and valence levels, which do not require focusing on the interpretation in relation to specific psychological states or emotions. Additionally, arousal and valence can be applied to the visualization on the emotion map, which is a tool that allows us to visually represent the emotional responses of individuals to different stimuli. By combining EEG and HRV measures with an emotion map, we can gain a more comprehensive understanding of the effects of olfactory stimuli on the emotional states of individuals.

The novelty of this research lies in its ability to provide a more nuanced and detailed picture of the psychological and emotional responses to olfactory stimuli. The emotion map can provide a clear visual representation of the specific emotions experienced by individuals in response to different scents. This information can help researchers identify patterns and relationships between different emotional states and specific olfactory stimuli. In addition, this research has practical applications in areas such as marketing and product design, by providing insights into the emotional responses of consumers to different olfactory products. It also has implications for the development of new treatments for mental health disorders that involve dysregulation of emotions and psychological responses. With a better understanding of the emotional effects of olfactory stimuli, there is a possibility to develop new therapies that can help regulate emotions and improve mental health outcomes.

However, it should be noted that our results are still limited. One limitation is that the EEG sensor can measure only the AF3 channel of the frontal lobe, and thus, we still cannot observe the asymmetry of EEG oscillations in the brain regions other than the frontal lobe. Even with such a limitation, we considered that our results are valid to some extent because the AF3 channel is at a close position to the olfactory bulb of the brain. Another limitation is that we used moving average as a smoothing method before calculating the EEG indexes. This method helps reduce noise and fluctuation in the signal, resulting in a clearer representation of the brain activity. However, it is important to note that applying this method can also result in an information loss in high-frequency bands such as theta and delta bands [60]. Therefore, other smoothing methods should also be considered, which remains as our future work.

## 7. Conclusions

In the modern information society, people are constantly exposed to stress due to complex work environments and various interpersonal relationships. Aromatherapy is attracting attention as one of the coping methods for relieving stress using aromas. Therefore, rather than going to a specialized aromatherapy shop, it is possible to do it easily at home. There are several methods of aromatherapy, and the easiest way is inhalation. Therefore, we consider that it is necessary to have a method that can evaluate the effect of aroma inhalation from the biological signal so that such an effect can be observed quantitatively and instantly.

In this study, 62 participants were recruited to conduct an aroma presentation experiment. The changes in biological signals (EEG and HRV) measured during aroma presentation were evaluated as the psychological response toward the olfactory stimuli. The experiment recruited male and female participants in their 20s to 50s equally. A total of seven stimuli were used, consisting of six aromas and one unpleasant odor. In the experiment, each stimulus was presented while measuring biological signals.

Next, we analyzed data by using the data grouped by gender and generation as well as stimulus exposure time. The results show a certain relationship between biological indexes and psychological states during some olfactory stimuli. From the overall observation of results, the psychological effects from olfactory stimuli can be observed more clearly during the first half of the stimulus presentation, even if the period is as short as 30 s. The results imply that humans have an immediate response to an olfactory stimulus before gradually adapting their body to a more neutral state. Moreover, we obtained the tendency of a stronger effect of aroma stimuli to the psychological states than unpleasant odor. Specifically, the results of male participants in their 20–30s show significant differences in EEG (i.e., Attention−Meditation and MA Hβ/Hα) and HRV (i.e., pNN50) indexes between aromas and unpleasant odor. On the other hand, the results of delta wave as EEG index and RMSSD as HRV are significantly different across genders and generations, which show their potential to generalize our method to evaluate psychological states influenced by olfactory stimuli. Furthermore, the results from the emotion maps suggest the appropriate range of EEG frequency bands for the detailed analysis of psychological states applied to the olfactory stimuli.

Our study contributes to the field of neuromarketing such as an evaluation of human psychological states toward aromatherapy products. Rather than medical-grade sensors, we employed commercially available and simple-to-use EEG and PPG sensors, and applied them to visualize an emotion map to provide a detailed picture of psychological states toward the olfactory stimuli. Our proposed method has potential to be further applied to neuromarketing research.

In conclusion, the results of this study suggest the possibility of using biological indexes to evaluate psychological states toward olfactory stimuli. In addition, it was confirmed that it is possible to visualize psychological states for olfactory stimuli using emotion maps. This result will be further analyzed by using multiple biological indexes in the future.

## Figures and Tables

**Table 1:** Olfactory stimuli used in the experiment.

Stimulus Name	Stimulus Type
Citrus	Aroma
Lavender	Aroma
Soap	Aroma
Woody	Aroma
Fruity	Aroma
Floral	Aroma
Unpleasant odor	Isovaleric acid (IVA)

**Table 2:** Number of participants divided by gender and generation.

Gender	Generation	Number of Participants
Male	20s	9
Female	20s	10
Male	30s	6
Female	30s	7
Male	40s	3
Female	40s	9
Male	50s	12
Female	50s	6

**Table 3:** EEG indexes and corresponding frequency bands and psychological states [50,51,52].

EEG Index	Frequency Band (Hz)	Related
Delta (δ)	1–3	Deep, dreamless sleep, unconscious
Theta (θ)	4–7	Intuitive, recall, relaxed meditative
Low alpha (α)	8–9	Relaxed but not drowsy, calm, eyes closed
High alpha (α)	10–12	Relaxed but not drowsy, conscious
Low beta (β)	13–17	Relaxed yet focused, integrated
High beta (β)	18–30	Thinking, aware of self and surroundings, alert
Gamma (γ)	31–50	Cognition, information processing, awareness

**Figure A1:**
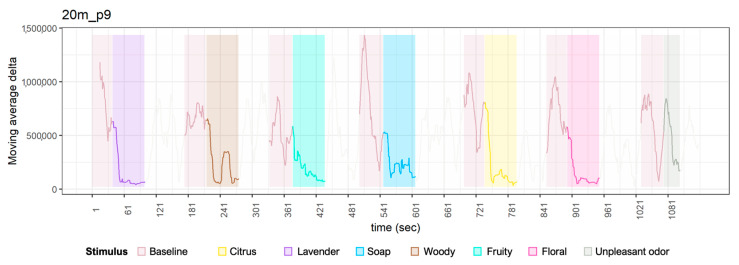
Time series plot of delta wave of one participant from the male 20s–30s group. The lines indicate the moving average values of delta wave Y-axis) against the time (X-axis) from the beginning to the end of the experiment. Different colors indicates different experiment conditions (olfactory stimuli or resting as baseline).

**Figure A2:**
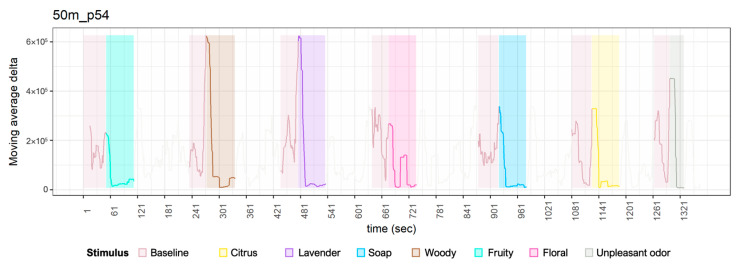
Time series plot of delta wave of one participant from the male 40s–50s group. The lines indicate the moving average values of delta wave Y-axis) against the time (X-axis) from the beginning to the end of the experiment. Different colors indicates different experiment conditions (olfactory stimuli or resting as baseline).

**Figure A3:**
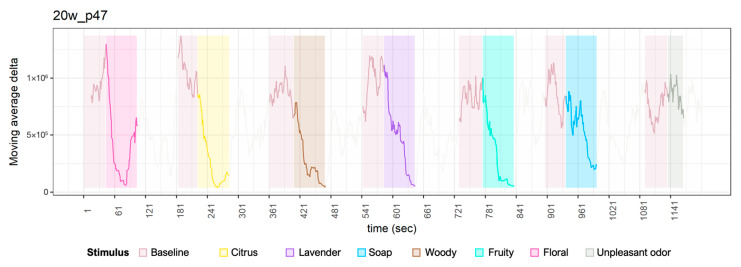
Time series plot of delta wave of one participant from the female 20s–30s group. The lines indicate the moving average values of delta wave Y-axis) against the time (X-axis) from the beginning to the end of the experiment. Different colors indicates different experiment conditions (olfactory stimuli or resting as baseline).

**Figure A4:**
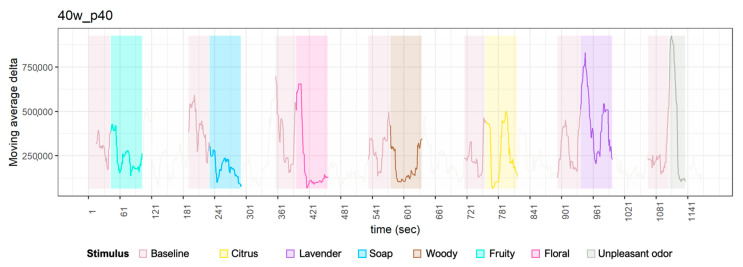
Time series plot of delta wave of one participant from the female 40s–50s group. The lines indicate the moving average values of delta wave Y-axis) against the time (X-axis) from the beginning to the end of the experiment. Different colors indicates different experiment conditions (olfactory stimuli or resting as baseline).

## Data Availability

The data presented in this study are available on request with a formal statement of reason and an intended purpose of use. The data are not publicly available due to ethical and privacy reasons.

## Abbreviations

EEG: electroencephalogram; HRV: heart rate variability; PPG: photoplethysmogram; ECG: electrocardiogram; ANS: autonomic nervous system; EDA: electrodermal activity; GSR: galvanic skin response; PET: positron emission tomography; IVA: isovaleric acid; δ: delta; θ: theta; α: alpha; β: beta; γ: gamma; Hβ/Hα: ratio of high beta and high alpha; MA: moving average; RMSSD: root mean square of successive differences between normal heartbeats; LAHV: low arousal and high valence; LALV: Low arousal and low valence.

## Appendix A. Time Series Plot of Delta Waves

This appendix provides time series plot of delta wave during the experiment. Different colors on the plot indicate different periods for different stimulus exposure. A total of four plots are provided for the four participant groups.
